# Core competency gaps among Somalia's disease surveillance workforce: a cross-sectional study

**DOI:** 10.11604/pamj.2026.54.9.50073

**Published:** 2026-05-13

**Authors:** Abdirahman Mohamed Abdullahi, Abdullahi Mohamed Mohamud, Marian Muse Osman, Mohamed Abdirahman Abdi, Gallad Dahir Hassan, Yusuf Hared Abdi, Saadaq Adan Hussein, Salad Ahmed Halane, Mohamed Abdinor Omar, Sahra Isse Mohamed

**Affiliations:** 1 Department of Health Emergencies, Federal Ministry of Health, Mogadishu, Somalia,; 2 Faculty of Medicine and Surgery, Jazeera University, Mogadishu, Somalia,; 3 Department of Family Health, Federal Ministry of Health, Mogadishu, Somalia,; 4 Somalia National Institute of Health (NIH), Federal Ministry of Health, Mogadishu, Somalia,; 5 Faculty of Medicine and Health Science, Jamhuriya University of Science and Technology, Mogadishu, Somalia,; 6 Faculty of Graduate Studies and Research, Somali National University, Mogadishu, Somalia,; 7 Faculty of Medicine and Health Science, Hormuud University, Mogadishu, Somalia,; 8 Department of School of Postgraduate Studies, Benadir University, Mogadishu, Somalia,; 9 Department of Public Health, Ministry of Health, Galmudug, Somalia

**Keywords:** Disease surveillance, epidemiological capacity, competency gaps, global health security, Somalia

## Abstract

**Introduction:**

sensitive disease surveillance is essential for global health security, yet fragile, conflict-affected settings like Somalia face critical human resource and capacity challenges. District and regional surveillance officers are frontline personnel whose competencies in epidemiology, outbreak investigation, and data analysis directly influence early detection, rapid response, and containment of public health threats.

**Methods:**

a descriptive cross-sectional study was conducted from 1^st^ to 30^th^ April 2025 among all 90 active surveillance officers (72 District Surveillance Officers, 18 Regional Surveillance Officers) across Somalia's Federal Member States. A structured, self-administered questionnaire assessed demographic characteristics, training history (IDSR, FETP, outbreak investigation, rapid response, scientific reporting), and self-reported competencies in core surveillance domains.

**Results:**

of 90 invited officers, 68 completed the survey (response rate 75.6%). Participants were predominantly male (86.8%) and aged 20-39 years (95.6%), with 95.6% holding bachelor's or master's degrees. IDSR training coverage was high (96.6%), yet only 10.3% of district officers participated in FETP. Outbreak investigation experience was highest for cholera (54.4%) and measles (47.1%), but low (<20%) for AFP, malaria, diphtheria, and respiratory infections. Technical skills in Excel were widespread (≥75%), whereas GIS (25%) and Power BI (<5%) proficiency were limited. Geographic distribution was uneven, with 27.9% of officers based in Banadir and underrepresentation in peripheral regions.

**Conclusion:**

critical gaps in advanced epidemiological skills, specialized outbreak experience, and workforce equity among Somali surveillance officers undermine national disease detection and response. Prioritizing surveillance officers on FETP, targeted refresher training, technical upskilling in GIS and data visualization, and motivation are recommended.

## Introduction

Robust disease surveillance systems constitute the fundamental cornerstone of global health security, enabling the early detection, rapid response, and effective containment of public health threats that transcend national boundaries [1,2]. District and regional surveillance officers serve as critical frontline personnel in these systems, functioning as essential human resources responsible for outbreak detection, epidemiological investigation, data collection and analysis, and coordinating timely public health interventions at local levels [3]. However, fragile health systems in conflict-affected settings face profound challenges that significantly undermine surveillance capacity, including severely limited financial and human resources, weakened healthcare infrastructure, ongoing insecurity that restricts field operations, and disrupted communication networks that impede timely disease reporting [4,5]. Somalia exemplifies these challenges, representing one of the world's most complex humanitarian contexts where persistent public health threats, including cholera, measles, malaria, COVID-19, and emerging infectious diseases, continue to cause substantial morbidity and mortality [6-8].

The country's surveillance system has been heavily dependent on the Integrated Disease Surveillance and Response (IDSR) strategy and Field Epidemiology Training Programs (FETPs) to build local capacity for outbreak preparedness and response [7,9]. These programs have trained a significant cadre of frontline responders, with over 80 FETP graduates from across all federal member States and nationwide IDSR training for health workers at different levels. Yet, the expected improvements in timely detection and effective response have not fully practiced, as evidenced by the persistent and recurring magnitude of disease outbreaks, with cholera outbreaks between 2017-2019 resulting in over 88,000 cases and 1,200 deaths, while measles outbreaks have affected thousands of children annually Despite these interventions, Somalia continues to experience recurring disease outbreaks, with cholera outbreaks between 2017-2019 resulting in over 88,000 cases and 1,200 deaths, while measles outbreaks have affected thousands of children annually [10,11]. The Electronic Early Warning and Response Network (EWARN) system, established during the 2017 cholera outbreak, has shown variable performance with significant gaps in geographical coverage and reporting completeness [5].

The persistence of these outbreaks reflects critical and varied gaps in human resource capacity, inadequate training coverage across surveillance personnel, and uneven geographic distribution of skilled epidemiologists throughout Somalia's regions [6,7]. FETPs have been recognized globally as essential capacity-building mechanisms that strengthen surveillance systems by training competent field epidemiologists capable of conducting outbreak investigations, surveillance system evaluations, and evidence-based public health responses [9,12]. However, limited evidence exists regarding the demographic characteristics, professional competencies, and training coverage of Somalia's surveillance workforce, particularly among District Surveillance Officers (DSOs) and Regional Surveillance Officers (RSOs) who form the backbone of the country's disease surveillance infrastructure [7]. Understanding the competency levels in core domains, including epidemiology, outbreak investigation, biostatistics, rapid response, and scientific communication, is essential for identifying capacity gaps and designing targeted interventions to strengthen Somalia's public health surveillance system [12,13].

Therefore, this study systematically characterizes the demographic and professional profiles of Somalia's district and regional surveillance officers, rigorously evaluates their competencies and training coverage in surveillance and outbreak response, and pinpoints critical gaps for targeted capacity strengthening. By generating robust, context-specific evidence, these findings will inform strategic workforce development, optimize training programs, and guide resource allocation to enhance Somalia´s ability to detect and contain public health threats in the country.

## Methods

**Study design and setting:** a descriptive cross-sectional study was conducted between April 5 and 17, 2025, among disease surveillance officers across 58 districts in five federal member States (Southwest, Galmudug, Hirshabelle, Jubaland, and the Banadir Regional Administration (BRA)). The cross-sectional design was selected as the most appropriate approach for assessing the current competency levels and training coverage of surveillance personnel at a specific point in time, enabling simultaneous measurement of demographic characteristics, professional qualifications, and self-reported competencies. Somalia's health surveillance system operates through a decentralized structure involving both regional and district-level officers responsible for disease surveillance, outbreak detection, and response coordination within their respective administrative jurisdictions.

**Study population and sampling:** the target population comprised all active public health surveillance officers working within Somalia's Integrated Disease Surveillance and Response (IDSR) system, totaling 90 officers, including 18 Regional Surveillance Officers (RSOs) and 72 District Surveillance Officers (DSOs). A census sampling approach was employed, inviting all eligible surveillance officers to participate in the study to maximize representativeness and ensure adequate power for subgroup analyses. Inclusion criteria were: 1) currently employed as a surveillance officer at the regional or district level; 2) actively involved in disease surveillance activities; and 3) willingness to provide informed consent for participation. Officers on extended leave, those with less than three months of service, or those unable to complete the questionnaire were excluded from the study.

**Data collection procedures:** data collection was conducted using a structured, self-administered questionnaire distributed electronically via KoBoToolbox, a widely used digital data collection platform validated for health research in resource-limited settings. The survey was introduced during a national outbreak investigation training workshop held at the Summer Hotel, Mogadishu, where the study objectives, procedures, and voluntary participation were explained to all participants. Following the orientation session, the questionnaire link was distributed electronically to all regional and district surveillance focal points across the country through official communication channels. The self-administration approach was chosen to minimize interviewer bias and allow participants to complete the survey at their convenience during the training period, thereby reducing potential recall bias and ensuring standardized data collection procedures.

**Data collection instrument:** a comprehensive structured questionnaire was developed specifically for this study, incorporating validated items from previous surveillance competency assessments and adapted to the Somali context. The instrument comprised four main sections: 1) demographic characteristics including age, gender, education level, and professional background; 2) employment details including job title, years of service, and regional assignment; 3) training history covering IDSR training, Field Epidemiology Training Program (FETP) participation, and specialized skill development; and 4) self-reported competencies in core surveillance domains including epidemiology, biostatistics, outbreak investigation, rapid response, laboratory coordination, and scientific communication. Response options utilize multiple-choice, Likert scales, and categorical variables to ensure comprehensive capture of relevant information while maintaining ease of completion.

**Data management and quality assurance:** all data were collected electronically through KoboToolbox and automatically stored in a secure, password-protected database with restricted access limited to the research team. Data quality assurance measures included real-time validation checks, mandatory field completion for key variables, and range restrictions to prevent data entry errors. Regular data backup procedures were implemented, and all collected data were exported to Microsoft Excel and subsequently imported into RStudio version 4.5.1 for statistical analysis. Data cleaning procedures included identification and resolution of duplicate entries, assessment of missing data patterns, and verification of logical consistency across related variables.

**Statistical analysis:** descriptive statistical analyses were performed using RStudio version 4.3.0 to characterize the study population and assess competency distributions. Continuous variables were summarized using means and standard deviations or medians and interquartile ranges, depending on data distribution, while categorical variables were presented as frequencies and percentages. Cross-tabulations were conducted to examine associations between demographic characteristics, training exposure, and competency levels across different job categories (DSO, RSO, and Others).

**Ethical considerations:** ethical approval for this study was obtained from the National Institute of Health (NIH) of the Federal Ministry of Health of Somalia prior to data collection initiation with reference to NIH/IRB/62/DEC/2024. All participants provided voluntary informed consent before completing the questionnaire, with a clear explanation of study objectives, procedures, potential risks and benefits, and the right to withdraw at any time without consequences. Participant confidentiality and anonymity were strictly maintained throughout all study procedures, with no personally identifiable information collected or stored. Data access was restricted to authorized research team members only, and all data handling procedures adhered to established ethical guidelines for health research in humanitarian settings.

## Results

**Participants:** Table 1 shows the descriptive summary of the demographic and professional profiles of the 68 surveillance officers who participated in the study. The table details their distribution across age groups, gender, highest education level, professional background, current job title, and years of experience in the field. Of the 90 invited officers eligible for the study, 68 of them completed the survey (response rate 75.6%). 51.5% were aged between 20-39 years, followed by 44 % (n=30) in the 20-29 years category. Males comprised the majority of participants (86.8%, n=59) compared to females (13.2%, n=9), reflecting the gender distribution typically observed in surveillance and public health workforce in similar settings [14]. Educational attainment was notably high, with 95.6% holding university-level qualifications, including 76.5% (n=52) with bachelor's degrees and 19.1% (n=13) with master's degrees, while only 4.4% had certificate or diploma-level education.

**Table 1:** demographic and professional characteristics of the surveillance officers in Somalia (N=68)

Characteristic	Category	N	Percentage (%)
**Age group**	20-29 years	30	44.1
	30-39 years	35	51.5
	40-49 years	2	2.9
	50+ years	1	1.5
**Gender**	Female	9	13.2
	Male	59	86.8
**Education level**	Bachelor’s degree	52	76.5
	Master’s degree	13	19.1
	PhD		
	Certificate	2	2.9
	Diploma	1	1.5
**Profession**	Nurse	19	27.9
	Epidemiologist	15	22.1
	Public health officer	14	20.6
	Doctor	7	10.3
	Others	6	8.9
	Lab technician	3	4.4
	Laboratory staff	2	2.9
	Veterinarian	2	2.9
**Job title**	DSO	58	85.3
	RSO	4	5.9
	Others	5	7.4
	DHO/DMO	1	1.5
**Years of experience**	2-3 years	32	47.1
	5+ years	22	32.4
	3-4 years	9	13.2
	Less than 1 year	5	7.4

PhD: Doctor of Philosophy, DSO: District Surveillance Officer, RSO: Regional Surveillance Officer, DHO: District Health Officer, DMO: District Medical Officer

**Professional characteristics:** the professional composition revealed a diverse multidisciplinary team with nurses representing the largest group (27.9%, n=19), followed by epidemiologists (22.1%, n=15) and public health officers (20.6%, n=14), collectively accounting for 70.6% of participants. Medical doctors comprised 10.3% (n=7) of the sample, while laboratory personnel (laboratory staff and technicians combined) represented 7.3% (n=5). Job title distribution was dominated by District Surveillance Officers (DSO) at 85.3% (n=58), with Regional Surveillance Officers (RSO) comprising 5.9% (n=4) and other positions accounting for 8.9% (n=6). Regarding professional experience, nearly half of the participants (47.1%, n=32) had 2-3 years of experience, while 32.4% (n=22) had five or more years of experience, indicating a workforce with substantial field experience in surveillance activities, as 79.5% had more than two years of professional experience, as shown in Table 1.

**Figure 1:**
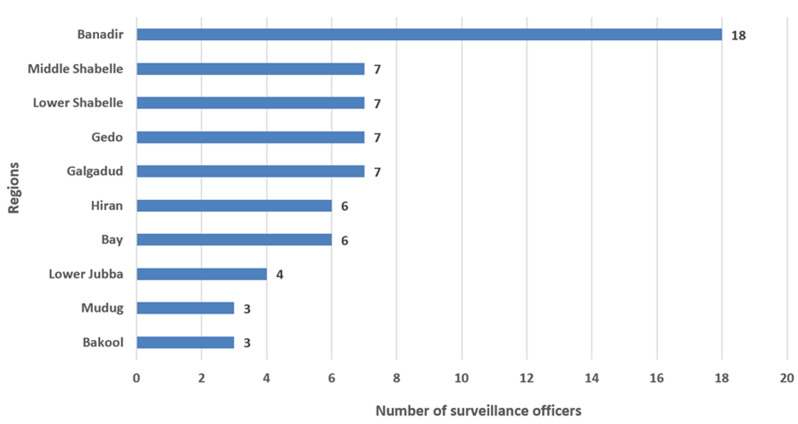
geographic distribution of surveillance officers across Somalia by region (N=68)

**Geographic distribution of surveillance officers:** Figure 1 illustrates the pronounced geographic disparities in the allocation of disease surveillance officers across Somali regions. It highlights the substantial centralization of capacity in the Banadir region (29.4%) and the underrepresentation in peripheral regions such as Bakool, Mudug, and Middle Shabeelle, underscoring potential vulnerabilities in early outbreak detection and response. In contrast, peripheral regions such as Bakool, Mudug, and Middle Shabelle have markedly fewer officers, with each comprising less than 5% of the total workforce (Figure 1).

**Training and the competency profile:** Table 2 compares the training history and participation in key programs among different surveillance officer cadres: District Surveillance Officers (DSOs), Regional Surveillance Officers (RSOs), and other staff. It details attendance of Integrated Disease Surveillance and Response (IDSR) training, participation in Field Epidemiology Training Programs (FETP), and other specialized training in outbreak investigation, rapid response, and scientific reporting. The majority of the District Surveillance Officers (DSOs) (96.6%, n=56) and all Regional Surveillance Officers (RSOs) (100%, n=4) received IDSR training. Among DSOs, 39.7% had received IDSR training within the past year, compared with 50.0% of Others and 100.0% of RSOs, while fewer DSOs (22.4%) had training two years prior, and none of the smaller groups did. Participation in the Field Epidemiology Training Programme was modest among DSOs (10.3%, n=6) but substantially higher for Others (50.0%, n=3) and RSOs (50.0%, n=2), with RSOs evenly split between Cohorts 3 and 5 and Others primarily in Cohort 6 (66.7%, n=2). Outbreak investigation training was widespread across all cadres (DSO: 75.9%, n=44; Other: 66.7%, n=4; RSO: 100.0%, n=4), whereas rapid response and scientific reporting training were less common and fairly uniform across positions (rapid response ~50.0%; scientific reporting ≤25.0%), indicating critical gaps in advanced epidemiological skills among surveillance personnel as shown in Table 2.

**Table 2:** training and the competency profile of the surveillance officers in Somalia (N=68)

Training type	Category	DSO; n (%)	Other; n (%)	RSO;n (%)
**IDSR training**				
Attended training	Yes	56 (96.6)	4 (66.7)	4 (100.0)
Last IDSR training	1 year ago	23 (39.7)	3 (50.0)	4 (100.0)
	2 years ago	13 (22.4)	0 (0.0)	0 (0.0)
	Last year	17 (29.3)	1 (16.7)	0 (0.0)
	More than 2 years ago	3 (5.2)	0 (0.0)	0 (0.0)
**FETP participation**				
Participated in FETP	Yes	6 (10.3)	3 (50.0)	2 (50.0)
	Cohort 1	0 (0.0)	0 (0.0)	0 (0.0)
FETP cohort	Cohort 2	1 (16.7)	0 (0.0)	0 (0.0)
	Cohort 3	0 (0.0)	1 (33.3)	1 (50.0)
	Cohort 1	0 (0.0)	0 (0.0)	0 (0.0)
	Cohort 5	2 (33.3)	0 (0.0)	1 (50.0)
	Cohort 6	3 (50.0)	2 (66.7)	0 (0.0)
**Other training types**				
Outbreak investigation	Yes	44 (75.9)	4 (66.7)	4 (100.0)
Rapid response	Yes	29 (50.0)	3 (50.0)	2 (50.0)
Scientific reporting	Yes	7 (12.1)	0 (0.0)	1 (25.0)

FETP: Field Epidemiology Training Program, IDSR: Integrated Disease Surveillance and Response

**Figure 2:**
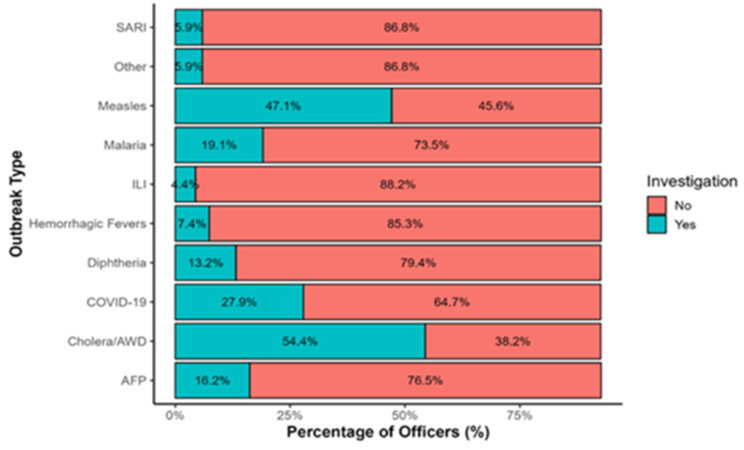
outbreak investigation experience by disease type among surveillance officers in Somalia (N=68)

**Outbreak investigation experience:** Figure 2 shows a bar chart showing the variation in officers' practical field experience in investigating specific disease outbreaks. It reveals substantial experience with cholera/acute watery diarrhea (AWD) and measles, but markedly limited investigation experience for acute flaccid paralysis (AFP), malaria, diphtheria, and respiratory infections, indicating critical capacity gaps. The study showed that surveillance officers had substantial variation in outbreak investigation experience across different disease types, with cholera/acute watery diarrhea (AWD) representing the most frequently investigated condition (54.4% of officers), followed by measles (47.1%) and COVID-19 (27.9%). Conversely, investigation experience was markedly limited for several priority diseases, with fewer than 20% of officers having investigated acute flaccid paralysis (AFP) (16.2%), malaria (19.1%), diphtheria (13.2%), hemorrhagic fevers (7.4%), or influenza-like illness (ILI) (4.4%). Notably, severe acute respiratory illness (SARI) and “Other” outbreak types showed the lowest investigation rates at 5.9% each, indicating significant gaps in practical field experience for respiratory and emerging disease outbreaks, as shown in Figure 2.

**Figure 3:**
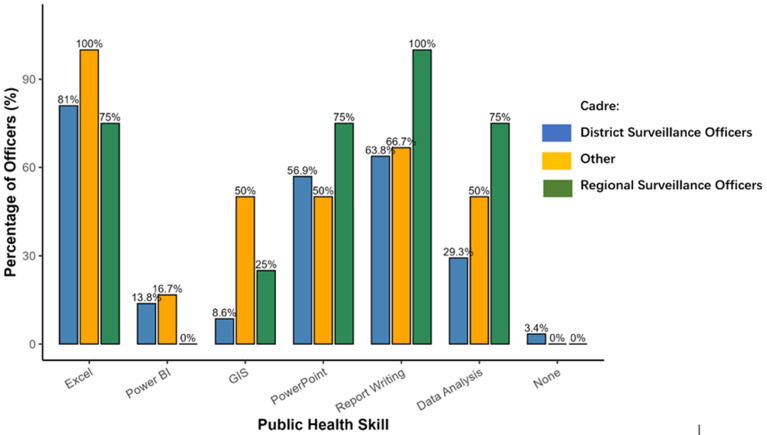
distribution of public health technical skills by job cadre (N=68)

**Public health technical skills:** Figure 3 shows a bar chart of proficiency levels in various technical skills (Excel, GIS, Data Analysis, and Power BI) across different job cadres. It shows widespread proficiency in Excel but limited skills with advanced data visualization and analysis tools such as Power BI and GIS, highlighting critical gaps in modern surveillance capabilities. Excel proficiency was the most prevalent skill across all surveillance officer cadres (DSO: 81%, Others: 100%, RSO: 75%), while Regional Surveillance Officers indicated superior competency in advanced skills such as GIS (25%) and data analysis (75%) compared to District Surveillance Officers. Notably, Power BI proficiency was extremely limited across all groups, and 3.4% of DSOs reported having no technical skills, highlighting critical gaps in modern data visualization capabilities essential for effective surveillance operations, as shown in Figure 3.

## Discussion

### Non-response could be due to the operational duties, access issues, or lack of personal interest in the subject matter

The study reveals that Somalia's surveillance workforce is predominantly young and male, with the majority holding a bachelor's or master's degree, indicative of a strong academic foundation that aligns with gaps previously documented in sub-Saharan Africa, where nursing and basic public health degrees predominate among frontline staff, yet advanced epidemiological qualifications remain inadequate [15,16]. Despite high levels of IDSR training coverage, participation in Field Epidemiology Training Programs (FETP) was modest, and exposure to advanced surveillance competencies such as scientific reporting, rapid outbreak analysis, and laboratory-liaison skills remained limited, paralleling studies from other fragile settings that report constrained opportunities for continuing professional development and specialized skill acquisition in surveillance cadres [15,17]. Notably, outbreak investigation experience was relatively robust in priority endemic diseases such as cholera and measles, while significant gaps persisted for acute flaccid paralysis (AFP), malaria, and respiratory infections, reflecting broader regional trends where practical field investigation is frequently concentrated in a limited disease spectrum and subject to diagnostic and logistical constraints [17,18].

Regional disparities in workforce deployment were apparent, with a preponderance of officers located in Banadir and core urban centers and comparatively fewer in peripheral or rural zones, a challenge corroborated by health workforce distribution assessments showing inequitable deployment as a persistent bottleneck for effective surveillance and timely outbreak response in resource-limited African settings [16,19,20]. The underrepresentation in several regions underscores potential vulnerabilities in Somalia´s early outbreak detection and response, emphasizing the need for equitable redistribution of skilled personnel to enhance both national and transboundary health security. While the academic qualification levels of Somali surveillance officers are commendably high, persistent gaps in practical experience, for example, investigating emerging infections or deploying advanced analytical tools, point to over-reliance on a small cadre of better-trained staff, raising concerns about sustainability, scalability, and resilience in the face of multiple or concurrent outbreaks [15,21].

The data in Figure 2 suggest that while surveillance officers have substantial experience with common endemic diseases such as cholera and measles, there are critical capacity gaps in investigating vaccine-preventable diseases, emerging infectious diseases, and complex outbreak scenarios that require specialized epidemiological skills and may pose greater public health threats. Comparisons with reports from across fragile and conflict-affected African countries affirm both the strengths and the limitations described in Somalia: chronic shortage of skilled epidemiologists, overburdening of nurse and public health officer roles, and the widespread challenge of building career pathways and retention mechanisms for health surveillance professionals [15,16,21]. By contrast, more mature health systems typically demonstrate stronger retention of epidemiologists, greater in-service training rates, and more even distribution of the surveillance workforce, enabling more comprehensive, rapid responses to diverse health threats [15,19].

The pronounced male predominance is observed in Somalia's surveillance workforce (Table 1). Male (86.8%) vs. female (13.2%) reflects pervasive gender inequities that characterize health systems across sub-Saharan Africa, where structural barriers, cultural norms, and systemic discrimination limit women's participation in technical and leadership roles within public health sectors [22]. This gender imbalance is particularly concerning given the critical role of surveillance officers in outbreak detection and response, as it potentially limits the diversity of perspectives, community engagement capabilities, and cultural competence necessary for effective population health interventions, especially in contexts where women's health issues and community-based surveillance may be better addressed by female professionals [23,24]. Evidence from fragile and conflict-affected settings demonstrates that male-dominated health workforces often struggle to adequately reach and serve vulnerable populations, including women and children who may face cultural barriers to accessing services provided predominantly by male professionals [24]. The underrepresentation of women in Somalia's surveillance system mirrors broader patterns documented across sub-Saharan Africa, where, despite women comprising the majority of healthcare workers globally, they remain significantly underrepresented in technical, leadership, and specialized epidemiological roles due to limited educational opportunities, cultural constraints, and institutional discrimination that perpetuate occupational segregation [25].

Addressing this gender disparity requires deliberate policy interventions, including targeted recruitment strategies, mentorship programs for women in epidemiology, flexible work arrangements that accommodate cultural and family responsibilities, and institutional reforms that promote gender equity in career advancement opportunities within Somalia's public health system [22]. The current gender imbalance not only represents a missed opportunity for leveraging women's unique contributions to community health and surveillance but also undermines the system's capacity to effectively serve diverse populations and achieve optimal outbreak preparedness and response outcomes.

Somalia´s fragile surveillance capacity poses a significant risk beyond its borders, as delayed detection and insufficient outbreak response can enable uncontrolled disease spread across the Horn of Africa and beyond [26]. Porous land borders with Kenya, Ethiopia, and Djibouti facilitate cross-border transmission, while migration, trade, and air travel link Somalia to the Gulf States and Europe, amplifying the potential for global exposure. These vulnerabilities undermine compliance with the International Health Regulations (IHR 2005), eroding collective security against emerging health threats [27]. Strengthening Somalia´s surveillance workforce through targeted training, equitable deployment, and enhanced technical competencies is therefore not only a national imperative but a critical contribution to regional and global health security, demanding urgent action and sustained international support.

Immediate actions should prioritize strengthening Somalia´s surveillance workforce through expanded Field Epidemiology Training Program (FETP) capacity and targeted upskilling in critical outbreak domains. The Ministry of Health must set concrete targets to enroll at least half of all District and Regional Surveillance Officers into advanced FETP modules within three years, accompanied by mandatory refresher courses every five years to sustain competencies. Specialized training tracks for acute flaccid paralysis, malaria, and respiratory infections should be developed and delivered via blended learning and mentorship pairings, ensuring novice investigators acquire hands-on experience alongside seasoned experts. To address regional inequities, an equitable deployment strategy must be enacted, including the creation of regional training hubs, hardship allowances, and professional incentives to attract qualified officers to underserved areas. Parallel efforts are needed to modernize technical skills: comprehensive digital literacy programs in advanced epidemiological software, data visualization platforms, and GIS tools should be rolled out in partnership with international agencies, providing access to licensed software and e-learning resources.

Over the medium term, policy reforms should establish clear career pathways and retention mechanisms for surveillance officers. Promotion criteria must integrate competency assessments, continuing education requirements, and performance-based incentives, with linkages to regional university partnerships offering advanced public health degrees. Gender-responsive recruitment strategies and mentorship networks should be instituted to achieve at least 40% female representation within a decade, complemented by flexible work arrangements that accommodate cultural and family responsibilities [28]. Laboratory-surveillance integration can be reinforced through joint training programs and standardized protocols for specimen handling and data sharing [29]. Community-based surveillance should be expanded by training community health workers and traditional healers in event-based reporting, enhancing early warning in remote zones. Finally, sustainable financing mechanisms, including dedicated national budget lines, public-private partnerships, and a surveillance workforce development fund, must be secured to ensure long-term support for these initiatives and the resilience of Somalia´s public health surveillance system.

## Conclusion

Critical gaps in advanced epidemiological skills, outbreak investigation, and data visualization capacities among Somali surveillance officers highlight the urgent need for training investments, equitable distribution, and technology integration. Addressing these weaknesses will enhance Somalia´s contribution to regional and global health security.

### What is known about this topic

- Disease surveillance in fragile and conflict-affected settings is often constrained by limited human resource capacity;
- Competencies of the surveillance officers are key determinants of effective IDSR implementation and early outbreak detection and response.

### What this study adds

- This study provides the first systematic competency assessment of Somalia's national disease surveillance workforce;
- It identifies priority skill gaps in advanced epidemiology, GIS, data visualization, and outbreak investigation.
